# Which curriculum components do medical students find most helpful for evaluating AI outputs?

**DOI:** 10.1186/s12909-025-06735-5

**Published:** 2025-02-06

**Authors:** William J. Waldock, George Lam, Ana Baptista, Risheka Walls, Amir H. Sam

**Affiliations:** Imperial College School of Medicine, Imperial College London, Charing Cross Campus, London, W6 8RP UK

**Keywords:** Large language models, Generative artificial intelligence, Undergraduate medical assessment, Case-based learning, Pathology

## Abstract

**Introduction:**

The risk and opportunity of Large Language Models (LLMs) in medical education both rest in their imitation of human communication. Future doctors working with generative artificial intelligence (AI) need to judge the value of any outputs from LLMs to safely direct the management of patients. We set out to investigate medical students’ ability to evaluate LLM responses to clinical vignettes, identify which prior learning they utilised to scrutinise the LLM answers, and assess their awareness of ‘clinical prompt engineering’.

**Methods:**

Final year medical students were asked in a survey to assess the accuracy of the answers provided by generative pre-trained transformer (GPT) 3.5 in response to ten clinical scenarios, five of which GPT 3.5 had answered incorrectly, and to identify which prior training enabled them to evaluate the GPT 3.5 output. A content analysis was conducted amongst 148 consenting medical students.

**Results:**

The median percentage of students who correctly evaluated the LLM output was 56%. Students reported interactive case-based and pathology teaching using questions to be the most helpful training provided by the medical school for evaluating AI outputs. Only 5% were familiar with the concept of ‘clinical prompt engineering’.

**Conclusion:**

Pathology and interactive case-based teaching using questions were the self-reported best training for medical students to safely interact with the outputs of LLMs. This study can inform the design of medical training for future doctors graduating into AI-enhanced health services.

## Practice points

Students reported pathology and interactive case-based teaching using questions as the best training to evaluate the LLM outputs.

Students need to be adequately equipped to engage with clinical prompt engineering to optimise the value of LLM outputs they interact with in their future practice.

## Introduction

Large language models (LLMs) are advanced probabilistic models of language [1], which are a form of artificial intelligence (AI) that can produce text output similar to human syntax. Generative pre-trained transformer (GPT) [2] is an LLM, which has demonstrated human-level performance on various professional and academic benchmarks [3]. Large language models appear to show promise in automating and delivering clinical processes, including passing accreditation exams [4]. This new capability prompts society to reflect on the added value of a doctor and how they should interact with LLMs when caring for patients. The supervisory role of doctors evaluating the value of LLMs is critical as they are developed for a range of administrative, diagnostic and management tasks. The risk and opportunity of LLMs in medical education both rest in their imitation of human communication, such as textbook paragraphs, and the replication of narrative knowledge, such as a curriculum. The breadth of LLM applications include clinical communication [5] and medical examination [6], including clinical assessment item writing [7].

There is great potential for LLMs to streamline the training of future doctors and staffing healthcare systems [8]. An LLM developed by Google called Med-PaLM 2 [9] has particularly drawn attention after achieving a convincing pass (85% accuracy) on the United States Medical Licensing Examination (USMLE), raising questions about the assessment of doctors and LLMs’ contributions beyond the retention of medical knowledge. Question-answer pairs for medical examinations are validated by experts and perceived as a legitimate substitute for real world training data written in narrative form, as per Google’s use of medical questions to develop Med-PaLM 2 [9]. The collection of established standard-set medical finals’ exam questions is therefore perceived to be a benchmark for evaluating LLM capabilities. However, large language models trained on written exams may lack nontechnical, nonverbal and contextual awareness to holistically evaluate real clinical scenarios [10].

In a cross-sectional multicentre study, most of the medical students perceived AI as an assistive technology that could facilitate physicians’ access to information, patients’ access to healthcare, and reduce errors [11]. They expressed their educational requirements as their need for knowledge and skills related to AI applications, applications for reducing medical errors, and training to prevent and solve ethical problems that might arise as a result of using AI applications [11]. We do not know the exact details for how AI will impact clinical practice, but we can aim to prepare students to be equipped to engage with LLMs. By improving AI literacy rates amongst future doctors, innovation may be accelerated for the benefit of patients and practitioners [12].

Clinical prompt engineering is the practice of composing carefully structured commands in the chat interface of an LLM to optimise the clinical utility of the output. This approach is essential for formulating inputs to an LLM and represents an important concept when assessing the safety and reliability of LLM-generated outputs. However, ‘hallucinations’ [13], variable performance of LLMs and the difficulty of reliable ‘clinical prompt engineering’ highlight the need for ongoing clinical supervision. ‘Hallucination’ of LLMs describes outputs which are incorrect or disconnected from the inputs. This leads to biased and convincing medical misinformation [14] with potential adverse consequences for patient care. Moreover, variability of LLMs when applied to electronic health records highlights the possibility of different responses to the same questions [15]. Large language models are curtailed by the initial pre-training text corpuses, which provide the underlying ‘learnt’ knowledge. When LLMs were evaluated for their ability to give therapeutic advice, distinct error patterns emerged including ambiguity and dangerous omissions [16]. Future medical graduates need to be able to interact with outputs of LLMs safely, possibly by using ‘clinical prompt engineering’ as the method of instructing an LLM to perform a clinically relevant task [17]. ‘Prompt engineering’ is a possible way to apply theoretical LLM knowledge to clinical scenarios, although this discipline currently remains unstable, with different clinical prompts being shown to have inconsistent effects in different LLMs [18]. If we are to become more reliant on AI, we need to equip students with the confidence to call out decisions made by AI.

This study aims to understand the learning activities, which final year medical students perceive to have informed their reviews of GPT-3.5 responses to medical school final exam questions. ‘Risk evaluation’ is defined in this context as the student’s ability to judge the value of the GPT’s answer for the safe care of the patient. Large language model clinical ‘risk evaluation’ is a multifaceted skill, which incorporates clinical reasoning ability, critical thinking, and awareness of the strengths and limitations of LLMs [19]. These challenges have highlighted the need for guidelines for the evaluation of decision support systems driven by AI [20] and tools for the quality assessment of diagnostic accuracy studies using AI (QUADAS)-AI [21]. The GMC’s ‘Outcomes for Graduates’ [22] defines the target outcomes for doctors graduating from UK medical schools and therefore directs the curation of medical curricula. Our study is of direct relevance to the GMC Outcomes ‘patient safety and quality’, ‘dealing with complexity and uncertainty’, and ‘using information effectively and safely’. We sought to explore medical students’ ability to validate LLM responses to clinical questions and understand which prior learning may equip them with the skills to make safe clinical decisions when AI is a potential source of information.

## Methods

The study received ethical approval from Imperial College London Education Ethics Review Process (EERP2324-018) and was carried out in accordance with the STROBE (Strengthening the Reporting of Observational Studies in Epidemiology) guidelines [23].

### Educational context and data collection

In preparation for summative assessments, Imperial College School of Medicine (ICSM) final year medical students received revision lectures in January 2024. We investigated the risk evaluation methodology of medical students responding to five questions about GPT 3.5 responses to previous final year exam questions as part of a revision lecture.

We asked GPT 3.5 to answer a set of single best answer (SBA) questions, each with five options. Final year students undertook a set of ten SBA questions, five of which had been answered correctly by GPT-3.5 (Table 1).

For each SBA question, students were asked the following questions:

- Did GPT-3.5 answer this question correctly?
- What information was required to determine the correctness of GPT-3.5’s response?

The questionnaire also included the following questions:

- Which areas of prior learning did you draw upon (e.g. experiential learning, specific medical school modules)?
- Which aspect of your medical school training was instrumental in analysing these responses?
- How might this knowledge inform a skill called ‘Clinical Prompt Engineering’?

Patient safety is related to both the outputs and the inputs of LLMs. The first two questions simulated a clinician interacting with the outputs of an LLM, assessing their ability to safely respond to an LLM’s clinical recommendations. The last question assessed a student’s awareness of clinical prompt engineering, the skill required to safely interact with the input of an LLM. This question is a necessary component of an investigation into LLM clinical safety, as it is the primary method by which a clinician will engage with LLMs. It is therefore an important part of assessing the educational needs of those who will interact with LLMs when caring for patients.

Students were prompted to answer each question individually and anonymously using Mentimeter, a cloud-based interactive presentation software [24]. They were then provided with immediate feedback on their responses as part of the revision lecture.

### Data analysis

The main corpus of analysis was constituted by the above questions, four of which were open-ended. To analyse textual data, we followed content analysis [25]. As the students answered the questions using Mentimeter, the responses were brief and ‘objective’. Students did not use sentences; mostly keywords and/or short expressions. This was therefore the unit of analysis that was used. We followed an inductive, conventional content analysis [26] as no previous framework had been established. Each keyword and/or expression were analysed only once into a code. Some codes were then broken down into more specific categories so we could try to elicit more meaning from the text produced by the students.

## Results

One hundred and forty-eight students gave consent for their responses to be part of this research project. The first question asked students if the GPT 3.5 response to the SBA question was correct (Table 1). Students’ justifications for their judgement were categorised as to whether they were ‘accurate’, ‘vague’ or ‘inaccurate’. The median percentage of students correctly determining the accuracy of the LLM output was 61%. When those who provided vague or inaccurate justifications were excluded, the median percentage of students who correctly evaluated the LLM output dropped to 56%. Further analysis of the accurate justifications revealed that 62% included a specific pathology test, 24% referred to a blood marker and 14% pointed to a specific symptom.

63% of students responded to the question regarding the areas of prior learning they drew upon to judge the LLM output. Students’ responses referred to medical school taught modules (46%), medical school online learning resources (21%), and external resources including online notes and question banks (33%).

57% of students answered the question about aspects of their medical school training, which they thought were instrumental in analysing the LLM outputs. 72% of the comments about the most valuable official medical school teaching for their evaluation of GPT 3.5-generated answers related to the pathology module or interactive case-based teaching sessions using questions.

50% of students answered the question about ‘clinical prompt engineering’, with only 5% knowing the concept. Those aware of ‘clinical prompt engineering’ were evenly split in designing prompts for specific clinical tasks and specifying GPT output requirements.

**Table 1:** Comparison of students’ responses across all ten clinical scenarios

Clinical presentation	GPT 3.5 Answer Correct	Response rate (%)	Students correctly determining LLM output accuracy (%)	Students correctly determining LLM output accuracy, but with vague/inaccurate justifications (%)
Breathlessness	No	98	80	14
Arrhythmia	No	86	83	15
Chest pain	Yes	78	59	23
Weight loss	No	72	71	20
Haemoptysis	Yes	67	64	14
Jaundice	Yes	64	56	25
Genital ulcer	Yes	60	57	2
Abdominal mass	No	62	63	4
Loss of vision	Yes	60	47	0
Haematuria	No	59	59	7

## Discussion

We identified a dominant theme of pathology-related teaching using questions in student perceptions of which prior learning activities equip them for engaging safely with LLM outputs. Pathology has been described as the ‘science underpinning medicine’ [27], and learning pathology remains important in training in all specialties and postgraduate assessments [28]. Pathology informs both diagnostic reasoning and therapeutic justification and is therefore integral to both the investigation and treatment of patients. Case-based learning using questions was reported by medical students as another aspect of their training that helped them to assess the LLM outputs. Our data show both formal and informal learning opportunities contribute to students’ ‘risk evaluation’ competency. Whilst former medical pedagogy was founded on textbooks as the main source of information for medical students and residents [29], the speed of change in AI presents profound challenges to the design of modern medical training. Of particular importance is the evolution of competency frameworks as a ‘series of propositions and relationships that collectively define an ideal’ [30], which must now be informed by the realities of AI-enhanced clinical practice [31]. The Canadian Medical Education Directives for Specialists (CanMEDS) Physician Competency Framework [32] describes the roles of a doctor as communicator, collaborator, leader, health advocate, scholar, professional, and medical expert. Changes brought about by AI affect all these roles [33].

Our results have corroborated the value of question based collaborative learning [34], the importance of question banks as a learning resource [35], and the value of questions in team-based learning [36], not least for higher enjoyment and engagement seen with interactive case-based learning amongst students [37]. The holistic integration of technical knowledge and sociocultural factors [38] are yet to be achieved by AI, but the patient-doctor relationship “has been altered into a triadic relationship by introducing the computer into the examination room” [39]. A proportion of students acknowledged the value of online question banks. An example of the application of questions to facilitate learning is ‘Question-Based Collaborative Learning’ in which students achieve their own constructive alignment of the curriculum and assessment by writing assessment items based on the patients they have seen [34].

AI literacy, of which LLM literacy is a subcomponent, has emerged in recent years as an essential skill within multiple disciplines and industries [40]. It is essential to develop AI-driven transformations, which respect the interest of patients. Students need to ascertain how to reliably judge if patient safety is threatened by the output of AI and be able to practise amongst this specific form of ‘complexity and uncertainty’. We need to develop robust pedagogy for scrutinising the factuality and bias of any outputs upon which clinical management is based. It is important to note that underlying bias from the socio-cultural context of the original text corpus will be retained in the LLM output. For example, Black and Hispanic patients who are not documented properly in electronic health records will not be represented appropriately in the development process of these LLMs, although natural language processes may be able to address this [41]. Therefore, medical education applications of LLMs must train doctors to supervise, evaluate and reflect on the value of any LLM output depending on the wider clinical context. This is essential for patient-centred, evidence-based medicine which engages with both the risks and benefits of LLMs. We need to incorporate AI literacy into clinical training, both at medical school and for postgraduate levels, with a particular focus on when patient safety concerns are triggered. A clinician should be able to flag when an LLM output does not fit the wider clinical picture and reject dangerous outputs. Collaboration between researchers, educators and practitioners is essential for developing transparent AI models that encourage the ethical and responsible use of AI in medical education [42].

A scoping review of literature from 2022 onwards about generative AI in the context of medical education identified three key areas for investigation: developing learners’ skills to evaluate AI critically, studying human-AI interactions and rethinking assessment methodology [43]. Our study also highlighted the shortage of medical student awareness of the essential skill of clinical prompt engineering. The students self-reported a lack of awareness of the concept, and do not therefore understand the concept as it relates to evaluating the clinical value of an LLM output. A previous study showed the majority of students and faculty learned about AI from the media [44], highlighting a vacuum of alternative reliable training. However, even when additional teaching programmes such as a five week ‘Introduction to Medical AI’ workshop were implemented for medical students, four challenges were identified: prior knowledge heterogeneity, attendance attrition, curricular design and knowledge retention [45].

Our study has several limitations. The response rate diminished as the questions progressed. Furthermore, not all areas of clinical practice were covered in the SBA questions, and vague answers from students compromised their representation in analysis. Furthermore, the survey did not allow us to explore the points raised by students in more detail. Future studies with student and staff focus groups could provide better insight into which aspects of the medical school curriculum equip students with the appropriate skills to evaluate the risks associated with AI-generated answers.

It is challenging to assess the baseline AI literacy of students, especially given the rapid advancements in LLM capabilities. AI literacy varies across institutions and is influenced by factors such as access to technology, prior exposure to AI tools and the integration of AI-related content into curricula. These variations make it difficult to generalise our findings to broader student populations. Furthermore, as LLMs continue to evolve, the skills required to interact with and critically evaluate their outputs could also change, potentially altering the relevance of our current findings.

To effectively appraise the answers provided by LLMs, physicians must first have an adequate baseline knowledge of the subject being assessed. In our study, 56% of the 148 participating students demonstrated this requisite knowledge. If students do not have the necessary pre-requisite knowledge in a given topic, they are not ready to engage in the highly sophisticated process of appraising LLM outputs in a safe and effective manner. Our study was conducted approximately eight weeks before the summative assessment window. Therefore, it is possible that participants would have performed better in the assessment following the completion of their revision for the summative assessments. Consequently, our findings may underrepresent the students’ ultimate potential to engage effectively with LLM outputs. Future studies should also include an inquiry into how interactive case-based learning using questions might help students to appraise LLM outputs specifically.

## Conclusion

AI is a sociotechnical reality, and we need to validate the pedagogical requirements for the next generation of doctors. Our study highlights the importance of pathology and interactive case-based teaching using questions when developing doctors who can practise safely amongst LLMs and other clinical decision support systems.

## Data Availability

All data relevant to the study are included in the manuscript.

## Abbreviations

LLM: Large Language Model
GPT: Generative Pre-trained Transformer
USMLE: United States Medical Licensing Examination
AI: Artificial Intelligence
QUADAS-AI: Quality Assessment of Diagnostic Accuracy Studies
GMC: General Medical Council
ICSM: Imperial College School of Medicine
SBA: Single Best Answer
CanMEDS: Canadian Medical Education Directives for Specialists
